# New Additions to the CRISPR Toolbox: CRISPR-*CLONInG* and CRISPR-*CLIP* for Donor Construction in Genome Editing

**DOI:** 10.1089/crispr.2019.0062

**Published:** 2020-04-21

**Authors:** Dorjee T.N. Shola, Chingwen Yang, Vhy-Shelta Kewaldar, Pradip Kar, Victor Bustos

**Affiliations:** ^1^ CRISPR and Genome Editing Resource Center, The Rockefeller University, New York, New York, USA.; ^2^ Laboratory of Molecular and Cellular Neuroscience, The Rockefeller University, New York, New York, USA.

## Abstract

CRISPR-Cas has proven to be the most versatile genetic tinkering system of our time, predominantly as a precision genome editing tool. Here, we demonstrate two additions to the repertoire of CRISPR's application for constructing donor DNA templates: CRISPR-*CLONInG* and CRISPR-*CLIP*. CRISPR-*CLONInG* (CRISPR-*C*utting and *L*igation *O*f *N*ucleic acid *In vitro* via *G*ibson) was devised to enable efficient cut-and-paste of multiple complex DNA fragments by using CRISPR-Cas9 as a digestion alternative with precision and exclusivity features, followed by joining the digested products via Gibson Assembly, to construct double-stranded DNA and adeno-associated virus (AAV) donor vectors rapidly without cloning scars. CRISPR-*CLIP* (CRISPR-*C*lipped *L*ong ssDNA via *I*ncising *P*lasmid) was devised as a DNA clipping tool to retrieve long single-stranded DNA (lssDNA) efficiently from plasmid, up to 3.5 kbase, which can be supplied as the donor template for creating genetically engineered mice via *Easi*-CRISPR. We utilized two different Cas types (Cpf1 and Cas9n) to induce two distinct incisions at the respective ends of the lssDNA cassette junctions on the plasmid, yielding three independent single-stranded DNA units of unique sizes eligible for strand separation, followed by target strand clip-out through gel extraction. The retrieval of the lssDNA donor circumvents involvements of restriction enzymes and DNA polymerase-based steps. Hence, it not only retains sequence fidelity but also carries virtually no restriction on sequence composition, further mitigating limitations on the current *Easi*-CRISPR method. With the add-on feature of universal DNA-tag sequences of Cpf1-Cas9 duo protospacer adjacent motif, CRISPR-*CLIP* can be facile and applicable to generate lssDNA templates for any genomic target of choice. Additionally, we demonstrate robust gene editing efficiencies in the neuroblastoma cell line, as well as in mice attained with the AAV and lssDNA donors constructed herein.

## Introduction

The versatility of class 2 CRISPR system is attributable to the simplicity of Cas nuclease being guided by a single programmable RNA^1^ coupled with a unique spacer sequence for precise target navigation. The commonly used CRISPR protein, SpCas9, recognizes a protospacer adjacent motif (PAM) NGG, which exists once in every 42 bases in the human genome.^2^ In addition, a mutant version from xCas9 group recognizes an even shorter PAM “NG,”^3^ along with Cpf1 for AT-rich sequences,^4^ these Cas variants further relax the PAM stringency to allow its binding to all four nucleotides of the DNA sequence. Together with Cas9's stability and ATP independent catalytic reaction for facilitating DNA cleavage,^5^ the CRISPR system has attracted a wide variety of applications, prevalently as a robust tool for precision genome engineering in mammalian cells.^6,7^

CRISPR-mediated genome editing is carried out by using RNA-guided Cas9 to induce a DNA break at the genomic locus of interest, followed by harnessing an innate DNA repair mechanism to create indels for variants of gene disruptions or genetic modifications with defined outcomes. The latter is attained by additionally supplying an exogenous DNA template that carries the desired sequence flanked by homology arms (HA) to CRISPR cut site(s), and through the homology-directed repair (HDR) pathway, desired modifications can be precisely integrated into the genome of target organisms in a highly efficient manner. To generate genetically engineered mouse models, donor templates can be supplied as either single-stranded oligodeoxynucleotides (ssODN) or double-stranded DNA (dsDNA) vectors.^8^ The former functions as an efficient donor in zygotes, yet imposes length limitations of ∼200 bases due to technical difficulties in chemical synthesis, making it suitable only for minor genetic alterations (<50 bp), whereas dsDNA-mediated editing can accommodate larger-scale genetic modifications (up to 10 kb or even longer), which has been routinely conducted via mouse embryonic stem cells (mESCs) due to poor efficiency in zygotes. Recent development of *Easi*-CRISPR (*E*fficient *a*dditions with *s*sDNA *i*nserts-CRISPR) has successfully expanded the use of single-stranded DNA donor up to ∼2 kbase long, referred to as long single-stranded DNA (lssDNA), to introduce modifications over much larger genomic regions in mouse and rat zygotes, as well as in human T cells.^9–11^ The length capacity of lssDNA suffices for most genome editing purposes that used to be mediated via the mESC route, whereby the timeline for generating the founder mice (F0) can be accelerated to as little as 2 months.

The construction of dsDNA donors commonly relies on BAC recombineering or multi-step cloning methods to ligate a vector backbone and multiple DNA fragments, which often need to be acquired from various existing plasmids through the use of polymerase chain reaction (PCR) amplification and restriction enzyme (RE). The development of seamless cloning methods, such as Gibson Assembly,^12^ allows multiple DNA components to be assembled into a custom donor vector in a single step *in vitro*, averting any footprint and incorrect insert orientation, offering a rapid alternative to the lengthy and laborious conventional approach for donor template assembly. Nonetheless, seamless cloning requires each of the assembly components to carry complementary overhang sites (e.g., type IIS RE sites and Gibson overhangs for Golden Gate and Gibson Assembly, respectively), which are routinely created by PCR amplification. Such a process tends to stumble over DNA sequences with extended length or complexity, a common scenario when amplifying vector backbones with highly repetitive or palindromic sequences (e.g., multiple Lox sites in FLEx vector or ITR sequences in adeno-associated virus [AAV] vector), hence hindering the vector cloning. To address these PCR issues, especially in acquiring vector backbones, we utilize CRISPR-Cas9 to replace RE as the digestion tool to excise undesired DNA segments from source vectors. Next, we designate Gibson Assembly over Golden Gate to facilitate seamless cloning because the former has the flexibility of only requiring overhang sites to present in either one of the DNA components to be assembled, instead of both. We term this strategy “CRISPR-*CLONInG*” (CRISPR-*C*utting and *L*igation *O*f *N*ucleic acid *In vitro* via *G*ibson). Despite RE long being a vital tool for digestion, there is a very slim chance of finding a suitable candidate that cuts at the target location not only precisely but also, more importantly, exclusively. While the technical concept of using CRISPR as the digestion tool for cloning purposes has been previously described in the CATCH method to manipulate bacterial chromosomal DNA,^13^ the CRISPR-*CLONInG* approach presented here nevertheless puts the said technique into the genome editing context particularly by demonstrating various modifications on plasmid DNA of specific targeting scheme to construct two commonly used donor vectors (FLEx and AAV) efficiently that used to represent fairly challenging molecular biology maneuverability in the mammalian genome editing system.

To generate lssDNA donors, the current methods reckon on either a PCR- or RE-based approach to procure the single-stranded templates from assembled dsDNA donors. Hence, the aforementioned technical challenges are categorically applicable. Here, we demonstrate another CRISPR-based strategy, termed “CRISPR-*CLIP*” (CRISPR-*C*lipped *L*ong ssDNA via *I*ncising *P*lasmid), to bypass involvements of PCR and RE for lssDNA donor generation.

## Methods

### CRISPR digestion of vector backbone for CRISPR-*CLONInG*

To form ctRNP complex of Cas9 (cat. # 1081060; IDT):crRNA (IDT):tracrRNA (cat. # 1072532; IDT) at a ratio of 1:2:2, 48.8 pmole of ctRNA (crRNA and tracrRNA) was added in an RNAse-free tube, heated at 100°C for 2 min, and cooled at room temperature (RT) for 10 min. Cas9 protein (2 μg) was added and incubated at 37°C for 10 min to form the ctRNP complex. About 2–3 μg of source DNA plasmid (FLEx and AAV vector; Addgene plasmid # 60229) was added with NEBuffer 3.1 (cat. # B7203S; NEB), DEPC ddH_2_O in a volume of 30 μL, and incubated at 37°C for >2 h. Cas9 was inactivated at 70°C for 15 min. ctRNA was degraded with 10 μg of RNase A (cat. #19101; Qiagen) at 37°C for 30 min and resolved on 0.9% agarose gel by electrophoresis, followed by gel extraction to acquire the vector of interest.

### PCR amplification of vector insert for CRISPR-*CLONInG*

All the primers (Supplementary Table S1) were ordered from IDT and Eurofins Genomics. Primer pair Neo-F and Neo-R was used to amplify FRT-Neo-FRT from pL451 plasmid,^14^ and tdTom-F and tdTom-R was used to amplify tdTomato gene from *Hex-tdTomato* plasmid (Hadjantonakis Lab., MSKCC). Primer pair AAV-F and AAV-R was used on custom gene synthesized dsDNA anchored in a default vector that carries 15 bp knock-in sequence flanked with two HA (∼400 bp each). Two different PCR systems were adopted: Herculase II Fusion DNA polymerase (part # 600679; Agilent) and the Accuprime Pfx DNA polymerase PCR system (cat. # 12344-024; Thermo Fisher Scientific). The PCR condition was 95°C for 3 min; 30 cycles of 95°C for 30 s, 60°C for 30 s, 72°C for 1 min/kb; and 72°C for 5 min. PCR-amplified DNA fragments were subjected to 0.9% agarose gel electrophoresis, and DNA of expected size were gel purified.

### Gibson (HiFi) Assembly for CRISPR-*CLONInG* and ligation reaction

The CRISPR digested vector backbones (∼50 ng for each FLEx and AAV) and PCR-amplified inserts carrying Gibson overhangs (FRT-Neo-FRT and tdTomato for FLEx; novel donor template for AAV) were assembled at a ratio of 1:2 with Gibson (HiFi) DNA Assembly Master Mix (cat. # E2621S; NEB) following the manufacturer's protocol. The reaction mix was incubated at 50°C for 1 h, and 2 μL of the assembled mix (∼5 ng of vector backbone) was transformed into competent cells (cat. # C3040H; NEB), followed by spreading on antibiotic-selective LB agar plates. Mini-prep (cat. # 27104; Qiagen) DNA was digested with appropriate RE(s) for diagnostic test followed by Sanger sequencing validation. Cloning of duo-guides for AAV-v2 assembly was via standard ligation reaction, using T4 DNA ligase (cat. # M0202; NEB) following the manufacturer's protocol.

### CRISPR-*CLIP*

The source plasmid where lssDNA will be retrieved from, as illustrated in Figure 4A, carries the lssDNA donor cassette (∼2.2 kb; containing 5′ HA + floxed exon 2 + 3′ HA) anchored in pUC57 (obtained via gene synthesis from Genewiz). The beginning of 5′ HA and toward the end of 3′ HA contain PAM sequences for Cas9 and Cpf1, respectively. Hence, the plasmid was digested at these two sites with Cas9n(D10A) (cat. #1081062; IDT) and Cpf1 (cat. # 10001272; IDT) accordingly. The RNA-guided Cas9 protein used for DNA incision was prepared at a ratio of 1:2:2 of protein:crRNA:trRNA. Specifically, gRNA for Cas9n (noted as ctRNA_Cas9n(D10A)_) was prepared by mixing 610 pmole of crRNA and 610 pmole of tracrRNA, whereas 625 pmole was used for Cpf1 gRNA (noted as crRNA_Cpf1_). Both gRNA were heated at 100°C for 2 min and cooled at RT for 10 min. Cas9n(D10A) and Cpf1 (50 μg each) were added into corresponding gRNA and incubated at 37°C for 10 min to form the protein–gRNA complexes, noted as ctRNP_Cas9n(D10A)_ (ctRNA_Cas9n(D10A)_ + Cas9n Protein) and cRNP_Cpf1_(crRNA_Cpf1_ + Cpf1 Protein), followed by gently mixing with donor plasmid (100 μg) with NEBuffer 3.1 and DEPC dH_2_O in a volume of 200 μL. The CRISPR digestion reaction was incubated at 37°C for at least 2 h or overnight for better DNA incisions.

Three incision-bearing plasmids (∼10 μg) was column purified (cat. # K220001; Invitrogen) to check for digestion. Then, 1, 2, and 3 μg of the eluate was mixed with three times the denaturing gel-loading buffer (DGLB; cat. # DS611; Diagnocine) and subjected to 70°C for 5 min, flash cooled on ice for 1 min, and resolved on 0.9% agarose gel by electrophoresis at a constant 100 V until the desired distance was attained. A double-digested sample by Cpf1 and WT Cas9 was also included as a control. Once the lssDNA of interest was separated, indicating successful digestion on the agarose gel that requires at least 30 min staining with EtBr (>0.5 μg/mL), the remaining 90 μg of the digested plasmid was DNA precipitated following the standard protocol. The 2 μg/lane, which gave the best separation, was scaled up for extraction using a QIAquick Gel Extraction Kit (cat. # 28704; Qiagen).

### Validation of lssDNA

The dsDNA plasmid (carrying donor cassette; ∼400 ng)^*^ was cleaved with ctRNP_Cas9(WT)_ and cRNP_Cpf1_ (∼3 pmole of protein and ∼6 pmole of gRNA) at 37°C for >2 h, followed by inactivation of protein and gRNA degradation following the same method described in the CRISPR-*CLONInG* section. In another replicate reaction, only an extra 20 units of BamHI (cat. # R0136S; NEB) was added. The CRISPR cleaved ± BamHI digested samples, along with lssDNA (∼200 ng)^‡^ ± BamHI, were subjected to 0.9% agarose gel electrophoresis at constant 100 V. The sense lssDNA (top strand) was sequenced with reverse primers.

### Gene editing in N2A cell line

N2A cell (Neuro-2a/Cas9-Rosa26-Neo; cat. # SL511; GeneCopoeia) was cultured in medium, comprising 44% Dulbecco's modified Eagle's Medium (cat. # 30-2002; ATCC), 50% Opti-MEM (cat. # 51985-034; Life Technologies) with 5% fetal bovine serum (cat. # 100-500; Gemini), and 1% penicillin-streptomycin (cat. # P4333; Millipore Sigma) in a 37°C humid incubator with 5% CO_2_. The AAV DJ serotype packaging was performed by a vendor (Vigene Biosciences) and obtained a titer of 1.7 × 10^13^ GC/mL. Overnight recombinant AAV (rAAV) infection at a ratio of 1:50 was used for optimal editing. The rAAV-infected cells were single-cell sorted using the BD FACSAria at our university's Flow Cytometry Resource Center and clonally expanded. Genomic DNA was extracted using a High Pure PCR Template Preparation Kit (ref. #11 796 828 001; Roche), and the Herculase II PCR system was used to amplify 1,352 bp of exon 10 mutant sequence, along with the neighboring sequence using the following TD PCR conditions: 95°C for 3 min; 10 cycles of 95°C for 15 s, 61°C for 20 s (−0.5/cycle), and 72°C for 1.5 min; 25 cycles of 95°C for 15 s, 56°C for 20 s, and 72°C for 1.5 min with primer pair (Psen1-F and Psen1-R; Supplementary Table S1), followed by Sanger sequencing read with Psen1-seq primer (Supplementary Table S1).

## Results

### CRISPR-*CLONInG* for construction of dsDNA and AAV donor vectors

#### Replacing undesired DNA segment on FLEx vector with new DNA inserts

**FIG. 1.**
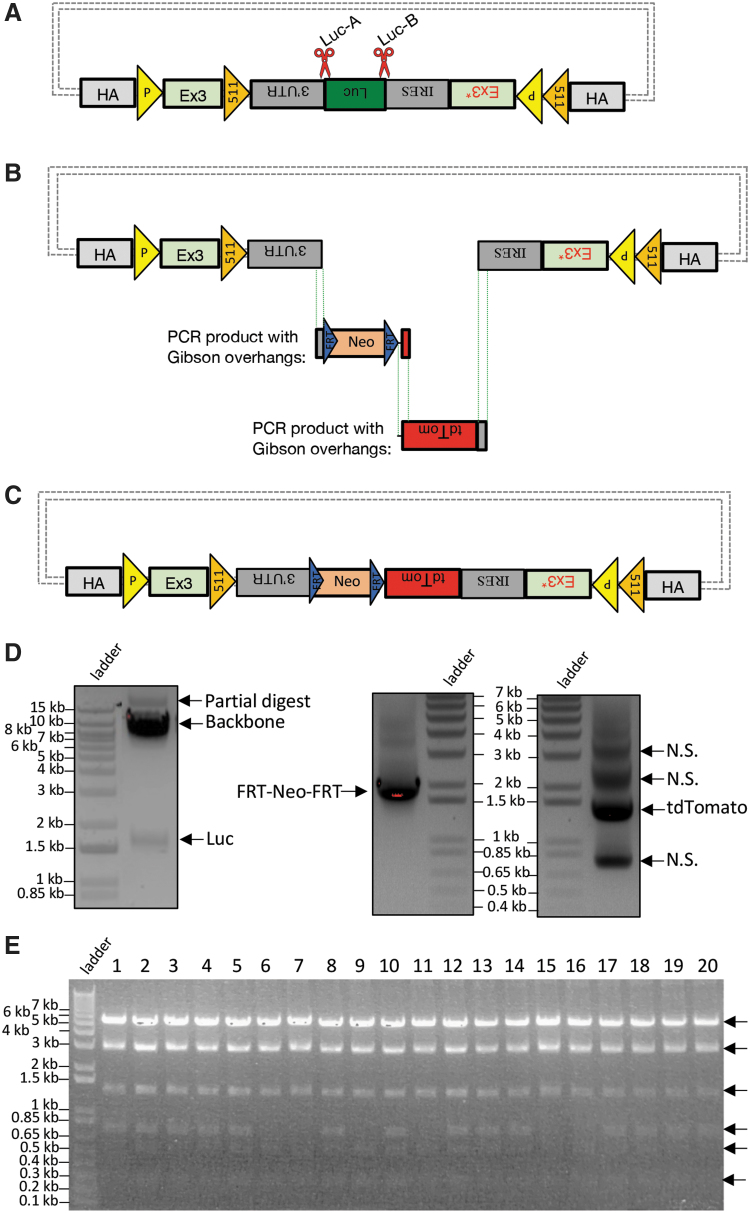
CRISPR-*CLONInG*: Replacement of Luciferase (Luc) on FLEx vector. **(A)** Schematic illustration of FLEx vector with CRISPR cut sites (red scissors) at the two junction sites flanking the undesired Luc fragment. Gray dot dashes: default backbone containing origin of replication and selection for propagation in bacterial host. **(B)** Luc was cut out with ctRNP (Cas9-ctRNA) complex; FRT-Neo-FRT and tdTomato were polymerase chain reaction (PCR)-amplified from existing plasmids using primers carrying complementary Gibson overhangs from the adjacent DNA fragment and vector backbone. **(C)** Two new vector inserts were joined with the CRISPR-digested backbone via Gibson (HiFi) Cloning for final donor assembly. **(D)** Excised FLEx vector backbone (∼7.5 kb) and Luciferase (∼1.65 kb; left); PCR amplified FRT-Neo-FRT (∼1.87 kb) and tdTomato (∼1.43 kb; right). N.S., nonspecific bands. **(E)** After CRISPR-*CLONInG*, 14/20 clones verified with PstI RE(s) diagnosis showed correct vector assembly (six DNA fragments; black arrow); three clones validated for sequence integrity. Resolved on 0.9% agarose gel.

To modify a FLEx (flip-excision) donor vector, we devised CRISPR-*CLONInG* to make customizations on the existing one. We proceeded by first using two gRNAs (Luc-A and Luc-B; Fig. 1A and Supplementary Table S2) with Cas9 (ctRNP complex) targeting the sites flanking the undesired Luciferase gene on the source FLEx vector for excision, rendering a backbone 7.5 kb long (Fig. 1D left) with complex sequences that were otherwise infeasible to acquire via inverse PCR amplification (data not shown). Next, we used PCR primers that carry corresponding 20–30 bp complementary Gibson overhangs to amplify the DNA sequences from two different plasmids for new vector inserts, FRT-Neo-FRT (∼1.87 kb) and tdTomato (∼1.43 kb; Fig. 1D right). The three DNA components thus acquired were joined together via Gibson Assembly (Fig. 1B and C) with a 70% success rate (Fig. 1E and Supplementary Data Sequence 1). The assembled FLEx vector was transfected into mESCs and successfully achieved target modifications (data not shown). The use of CRISPR-Cas9 allows the excision of the Luciferase gene from the existing vector to take place exclusively and precisely at its junction sites flanked by 3′-UTR and IRES sequences (Fig. 1A), with only 1 nt deviation, which can be easily remedied by an extra 1 nt carried in the primer overhangs (Supplementary Data Sequence 2).

#### Replacing cargo sequences on AAV vector with duo-guides and gene replacement donor

Viral delivery systems serve as potent gene delivery vehicles and have been the cornerstone of gene therapy. The AAV system is preferred over other viruses due to its nominal level of adverse immunogenicity in humans.^15^ In addition, AAV has been harnessed for targeted gene modifications (in contrast to adeno- and lenti-virus systems for transgenic purpose)^16^ long before the advent of programmable nucleases, primarily in somatic cell lines, due to its capability to transduce its cargo DNA effectively into cells that typically bear poor recombination efficiency for facilitating gene targeting.^17,18^ Incorporating AAV delivery with the CRISPR system has synergized the capacity of genome engineering to manipulate genetic contexts effectively in adult mice, such that disease models could be rapidly generated within several weeks, ready for biomedical studies.^19,20^ While viral vector backbones are equipped with multiple cloning sites (MCS) for cargo sequence engineering, suitable MCS scarcely exist for cargos with extended length, which is often the case in genome editing, or in the case of making modifications on the vector devoid of MCS, and inverse PCR cannot be an option. Hence, an alternative to the RE-based approach is crucially needed to facilitate efficient cut-and-paste of DNA segments on an AAV vector.

**FIG. 2.**
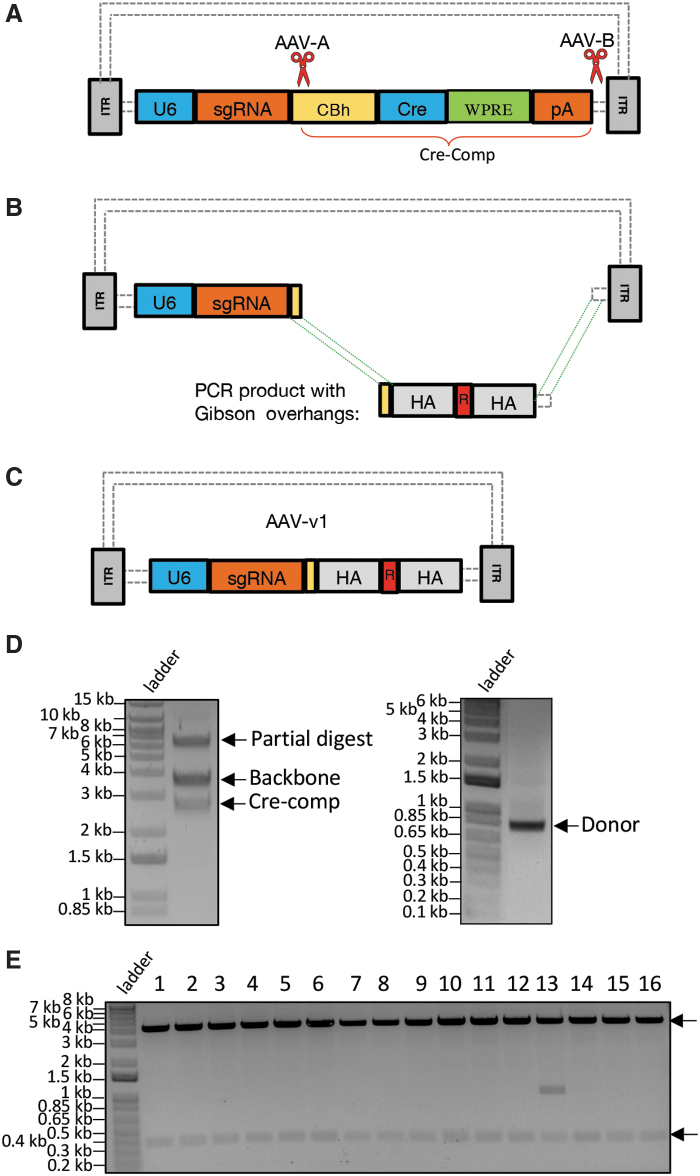
CRISPR-*CLONInG*: Replacement of partial cargo sequence (Cre-comp) with the desired donor sequence on adeno-associated virus (AAV) vector (#60229; Addgene). **(A)** Schematic illustration of AAV vector with CRISPR cut sites (red scissors) at two ends of the Cre-comp segment. Guides (AAV-A and AAV-B) with high on-target scores were selected. **(B)** Cre-comp was cut out with ctRNP (Cas9-ctRNA) complex; donor for gene replacement (containing 15 bp AA replacement sequence, noted as “R,” sandwiched by HA) flanked with complementary Gibson overhangs of the adjacent AAV backbone was PCR-amplified from custom gene synthesized plasmid. **(C)** Assembled AAV-v1: donor template cloned into the customized AAV backbone via Gibson (HiFi) Assembly. **(D)** Excised AAV vector backbone (∼3.63 kb) and Cre-comp (∼2.73 kb; left); PCR amplified donor template (∼0.8 kb) with Gibson overhangs (right). **(E)** After CRISPR-*CLONInG*, 15/16 clones showed correct vector assembly, confirmed by BbsI RE(s) diagnosis (two DNA fragments; black arrow); three clones further validated by Sanger sequencing. Resolved on 0.9% agarose gel.

Our target gene modification goal is to introduce three out of five amino acid (AA) changes within a 15 bp range (Supplementary Data Sequence 3) in the exon 10 region of the *Psen1* gene in neuroblastoma cell line (N2A). To construct an AAV vector to suit this purpose, we customized an existing one in two steps to replace two pieces of partial DNA cargos with desired sequences encoding CRISPR duo-guides and a donor template, respectively. The latter carries 15 bp AA replacement sequence flanked by HA to the target genomic site. The source AAV vector consists of a U6-driven guide with single guide RNA (sgRNA) cloning site, plus Cre and other components (noted as Cre-Comp). At the first stage of vector customization, we devised CRISPR-*CLONInG* using two gRNAs (AAV-A and AAV-B; Fig. 2A and Supplementary Table S2) with Cas9 (ctRNP complex) to excise Cre-Comp from the AAV backbone (Fig. 2A and D left), followed by PCR primers to amplify a custom gene synthesized plasmid that carries ∼0.8 kb donor template flanked with 30–35 bp Gibson overhangs (Fig. 2D right). The donor sequence was ligated into the newly modified AAV backbone through Gibson Assembly (Fig. 2B and C) with >90% cloning efficiency (Fig. 2E).

**FIG. 3.**
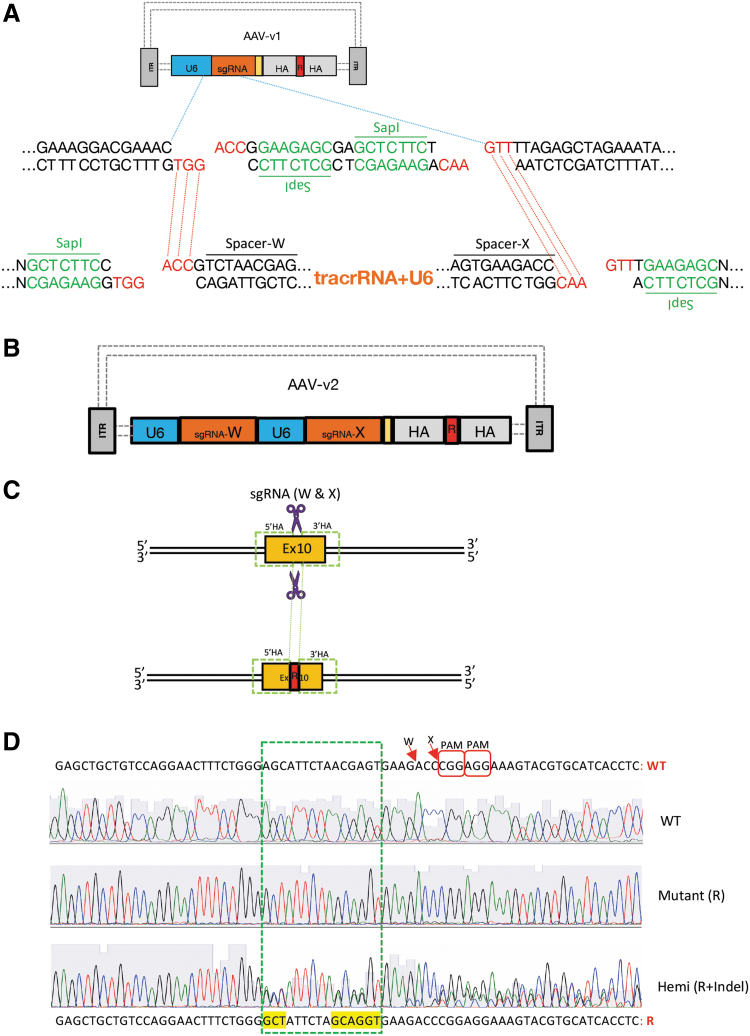
Cloning duo-guides into AAV-v1 vector by exploiting built-in cloning site (originally designed for one single guide RNA [sgRNA]). Gene editing (amino acid [AA] changes at *Psen1* gene) in N2A cell line. **(A)** Top: Schematic AAV-v1 construct: blue dotted line zooming out partial sequences of U6 and sgRNA cloning site. Middle: Type IIS RE (SapI) digest creates two unique 5′ overhangs (GGT vs. GTT). Bottom: Showing gene synthesized plasmid carrying duo-sgRNA cassette sequence (Spacer-W + tracrRNA + U6 + spacer-X) with complementary overhangs, flanked by uniquely positioned SapI sites, which upon SapI digestion renders two complementary 5′ overhangs (ACC vs. AAC; red dotted line) for cloning. **(B)** Schematic of the final construct AAV-v2. **(C)** Top: CRISPR guides (W and X) target sites (purple scissors) on Psen1 exon 10 region. Bottom: AA replacement donor (R) integrated at the genomic target after rAAV-v2 transduction. **(D)** Psen1 exon 10 sequence shown (WT vs. R: 3/5 AA changes—three amino acid codons yellow highlighted). Red arrow: SpCas9 guides (W and X) cutting sites; chromatograms showing WT, mutant (R) and hemizygous (R + indel); green dotted rectangle encompassing the 3/5 AA changes within a 15 nt range.

We further customized the assembled AAV vector (referred to as AAV-v1) to accommodate two CRISPR guides (sgRNA-X and sgRNA-W; Fig. 3B and Supplementary Data Sequence 3) targeting the exon 10 sites that span the AA change region. To exploit AAV-v1's built-in cloning site for a single sgRNA, which upon double digestions with type-IIS enzyme (SapI), two unique 5′ overhangs (GGT on the bottom strand and GTT on the top strand) were created (Fig. 3A middle). We designed a 0.5 kb gene synthesized plasmid to carry duo-sgRNA cassette (spacer-W + tracrRNA + U6 + spacer-X) with matching overhangs, plus two uniquely positioned SapI recognition sites flanking both ends. Specifically, one SapI recognition site was placed on the top strand 1 nt further adjacent to the overhang complementary to 5′-GGT, whereas another SapI recognition site was placed on the bottom strand 1 nt further adjacent to the overhang complementary to 5′-GTT (Fig. 3A bottom). Upon digestions by two SapI, the aforesaid plasmid rendered two overhangs complementary to the original sgRNA cloning site on AAV-v1, which then was ligated into AAV-v1 via type-IIS RE-based cloning whereby the duo-guides were engineered into the final AAV construct (referred to as AAV-v2; Fig. 3B). The AAV-v2 was used for viral packaging to produce the recombinant AAV, which subsequently was used to infect Cas9-expressing N2A cell line and successfully introduced its two-piece cargo into the targets, achieving desired AA changes (Fig. 3C). Robust gene editing efficiency was observed, with ∼5% screened clones (2/46 verified by Sanger sequencing) showing bi-allelic knock-in (Fig. 3D), 30% (14/46) with hemizygous knock-in, and the remaining 65% with indels (data not shown).

### CRISPR-*CLIP* for procuration of lssDNA donor

Various approaches have been proposed to construct lssDNA templates since the introduction of *Easi*-CRISPR, including *iv*TRT (*in vitro* transcription and reverse transcription) from the original *Easi*-CRISPR protocol^21^; the dsDNA plasmid-retrieval-based method using RE (BioDynamics Laboratory kit); the PCR-based method, which uses a phosphorylated primer to label the undesired DNA strand for degradation (Takara Bio kit); and chemical synthesis by commercial vendors (e.g., Megamer by IDT). These methods enable the procuration of lssDNA with sequence fidelity (except *iv*TRT) and length extension up to ∼2 kbase. Certain constraints, however, could arise from the use of RE (efficacy, availability, or unintended RE cut on the donor sequence), as well as from technical difficulties in PCR-based or chemical synthesis (e.g., sequences with unusual repeats or specific nucleotides composed in too high/low percentage) to procure complex sequences, thus failing the lssDNA construct. Alternatively, we devised CRISPR-*CLIP* here to avoid such pitfalls.

**FIG. 4.**
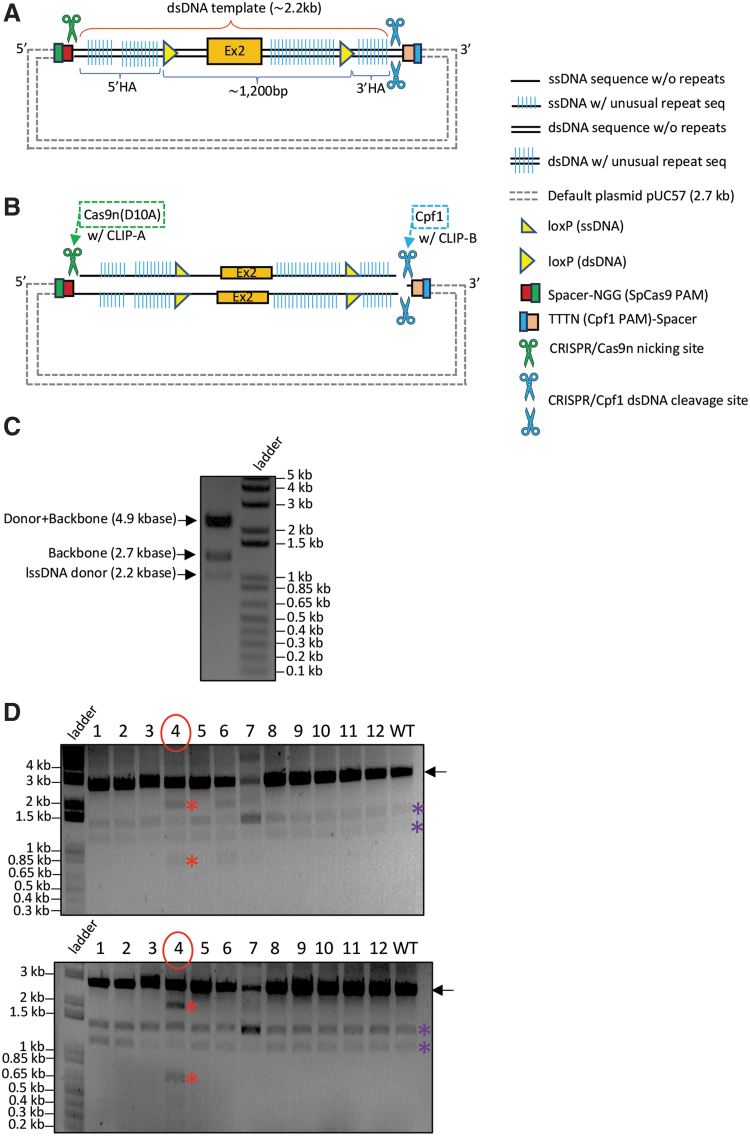
CRISPR-*CLIP*: Procuration of lssDNA from dsDNA template and genotyping results of the CKO mice generated with the acquired lssDNA via *Easi-CRISPR*. **(A)** dsDNA template anchored in the default plasmid; the sense ssDNA (top strand) is the donor (lssDNA) of choice. **(B)** Cpf1 (with guide CLIP-B) was used to create a dsDNA incision on the plasmid at one end of the lssDNA cassette, while Cas9n (with guide CLIP-A) was used to create a ssDNA incision at the other end, specifically on the strand of interest (top strand in this case). **(C)** Upon denaturing gel-loading buffer (DGLB) treatment, the plasmid incised by Cpf1 and Cas9n was resolved into three stand-alone distinct-sized units (0.9% agarose gel electrophoresis): ∼4.9 kbase (donor + backbone) vs. ∼2.7 kbase (backbone) vs. ∼2.2 kbase (lssDNA donor). **(D)** Mice genotyping screened by RE HindIII (top) and EcoRV (bottom): a pair of external screening primers amplified 2.8 kb DNA fragment (black arrow); mice with the lssDNA donor integration should carry a floxed cassette with HindIII/EcoRV site adjacent to LoxP. Upon RE digestion, mouse #4 showing the insertion of both LoxPs (∼0.8 kb vs. ∼2 kb; red asterisk), further confirmed by Sanger sequencing; mouse #6 showing only HindIII digest, indicating one LoxP insertion; mouse #7 was found to carry a heterozygous 1.2 kb deletion between the two guides (used for creating CKO model), verified by Sanger sequencing, thus showing a band at ∼1.6 kb. Due to incomplete RE digest and usage of EtBr pre-stained gel, the smaller bands appeared in lighter intensity; purple asterisk: non-specific PCR band.

To generate the lssDNA donor for creating a conditional knockout (CKO) mouse model of GENE-Y^†^ via *Easi*-CRISPR, we first obtained the gene synthesized dsDNA template, which consists of a floxed cassette of exon 2, flanked by HA to the genomic target. The template is 2.2 kb long and anchored in a default plasmid (pUC57; Fig. 4A). As the HA regions of the donor template encompass sequences with various types of unusual repeats that failed the lssDNA procuration via the PCR-based method (Takara Bio kit; data not shown), CRISPR-*CLIP* was adopted instead. We used Cpf1 and Cas9n (D10A mutant nickase version of Cas9)^1,7,22^ with corresponding guides to induce a dsDNA cleavage and a nick, respectively, on the plasmid at two junction sites flanking the lssDNA cassette. Specifically, Cas9n was exerted on the strand of interest (lssDNA; guides CLIP-B and CLIP-A; Fig. 4B and Supplementary Table S2). The resulting three stand-alone single-stranded DNA units were of unique sizes and hence were able to be separated using agarose gel electrophoresis upon DGLB treatment (Fig. 4C). The 2.2 kbase target strand of interest (i.e., lssDNA donor) was thus identified and clipped out through the gel extraction procedure.

**FIG. 5.**
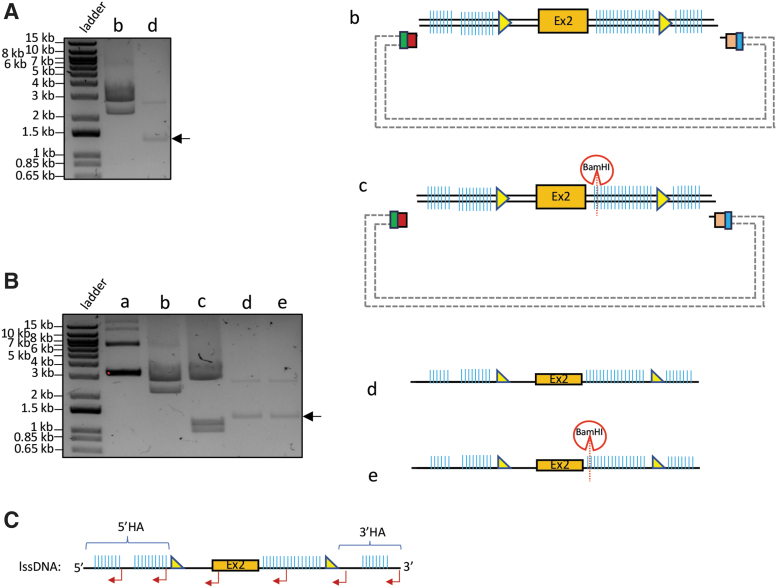
Validation of lssDNA acquired by CRISPR-*CLIP*. **(A)** Cpf1 and wild-type (WT) Cas9 digested the entire plasmid (∼4.9 kb, as shown in Fig. 4A) into ∼2.7 kb and ∼2.2 kb DNA fragments (lane b); acquired lssDNA (∼2.2 kbase) resolved around 1.2–1.3 kb in size^$^ (lane d, arrow). **(B)** Control: the uncut plasmid (lane a); control: as described in (A) (lane b); Cpf1 and WT Cas9, along with additional BamHI digested the plasmid into three fragments: BamHI cleaved the ∼2.2 kb dsDNA template into ∼1.2 kb and ∼1 kb, while ∼2.7 kb default plasmid remained intact (lane c); BamHI digestion did not cleave the lssDNA despite bearing a BamHI cut site (lane e, arrow)^$^ vs. lssDNA without BamHI digestion (lane d)^$^; all resolved on 0.9% agarose gel by electrophoresis. **(C)** Multiple reverse primers (red bent arrow) were used for Sanger sequencing validation of the acquired lssDNA (sense DNA, in this case). ^$^Due to the unstable nature of single-stranded DNA, the acquired lssDNA may not resolve at the exact predicted size (1.1 kb in this case) or a fraction could migrate at sporadic sizes on the agarose gel (as seen in the extra band above the desired band), depending on certain factors, such as buffer condition and potential secondary structure formation attributable to sequence composition, in which case treating the acquired lssDNA with DGLB followed by agarose gel electrophoresis could aid in size separation with more precise outcomes. The integrity of the procured lssDNA is considered of good quality as long as the majority of the lssDNA resolved close to the predicted size.

The lssDNA acquired was subjected to validations for intactness, single-strand feature, and sequence integrity. To verify length intactness, the 2.2 kbase lssDNA was resolved on gel by electrophoresis in parallel to its counterpart of source dsDNA (2.2 kb) for size comparison. To obtain the latter as a control reference, we used WT Cas9 and Cpf1 to digest the lssDNA-carrying plasmid (comprising 2.2 kb dsDNA version of template plus 2.7 kb pUC57 backbone, leading to 4.9 kb in total length), which yielded two DNA fragments migrating at respective sizes on the agarose gel (Fig. 5A, lane b), whereas the 2.2 kbase lssDNA donor migrated in the vicinity of 1.1 kb, indicating intact length (Fig. 5A, lane d). The single-stranded feature of the donor was verified by using dsDNA-specific RE BamHI to digest the lssDNA that carries a BamHI cut site. Upon BamHI digestion (in addition to WT Cas9 and Cpf1), the 4.9 kb lssDNA source plasmid, serving as a positive control, resulted in three digested fragments (Fig. 5B, lane c), while the acquired lssDNA did not yield any digestion product (Fig. 5B, lane e). Lastly, the sequence integrity was validated by Sanger sequencing using complementary primers (Fig. 5C). As a negative control, non-complementary primers were used to sequence the lssDNA and failed to pick up a correct reading, further reflecting the lssDNA purity (not contaminated with dsDNA). The lssDNA yield of 50–60% was obtained (e.g., digesting 100 μg of 2.2 kb dsDNA in 2.7 kb pUC57 backbone would result in >10 μg of lssDNA). The lssDNA thus procured was supplied as the donor into mouse zygotes via pronuclear microinjection and successfully generated CKO mice at 8% targeting efficiency (1/12 founder mice showed both LoxP inserted; Fig. 4D).

## Discussion

The two CRISPR-based methods shown in the above three cases offer effective strategies to overcome technical challenges frequently encountered in constructing donor templates for genome editing. Preferring to avert amplification issues with PCR, the CRISPR-*CLONInG* can be adjusted to utilize CRISPR-Cas9 as the excision tool to acquire every component of the vector, rather than only the backbone. In that case, the requisite Gibson overhangs for facilitating seamless cloning can be restored by additionally supplying a short gBlock carrying complementary sequences to the backbone–insert or insert–insert junction for Gibson Assembly reaction. Although the CRISPR-*CLONInG* introduces RNA components into the reaction, which could make the method seemingly difficult compared to the RE-based, considering that CRISPR technology has been adopted as the “go-to” tool for genome editing, where design and formation of ctRNP (gRNA-Cas protein) complex is a part of the experiment, it might not be an extra burden for end users to apply it for cloning purpose. Moreover, in terms of the material cost for each CRISPR-*CLONInG* reaction (e.g., as shown in Fig. 1A–C or Fig. 2A–C), the major expense (∼$170–190; IDT pricing) arises from the ctRNP complex: two crRNA (2 nM) are target specific (cost ∼$160–180), whereas tracrRNA and Cas9 protein are universal components that can be purchased in bulk, thus bringing the cost down to less than $10 per reaction. More importantly, the CRISPR-*CLONInG* offers flexibilities to construct highly customizable donor vectors, with guaranteed success within a few days. In contrast to adopting the conventional RE-based method for comparable tasks, which predisposes cloning scars and practically involves a more technically challenging procedure or which is simply not feasible, as described in previous sections, the CRISPR-*CLONInG* is a more cost-efficient option, especially when working with vectors with complex sequences.

The CRISPR-*CLIP* demonstrates a PCR-free-and-RE-free strategy that imposes no restrictions on sequence composition for procuring lssDNA donor templates. While the lssDNA yield thus obtained tends to be less efficient than the PCR-based approach, the resulting quantity is more than sufficient for multiple rounds of zygote microinjection to produce positive founder mice. For example, in the case of GENE-Y presented, digesting 100 μg input of a 4.9 kb source plasmid would result in 45 μg of 2.2 kb dsDNA, which upon gel extraction typically recovers 50–60% of the DNA, thus yielding 11–13 μg of the single-stranded output (i.e., 2.2 kbase lssDNA donor). For each round of mouse zygote injection (which involves 100–150 zygotes), we routinely prepare 100 μL of CRISPR reagent mixture containing 10 ng/μL per kbase in length of the lssDNA template. Hence, the required amount for the 2.2 kbase lssDNA is as little as 2 μg/100 μL. Obtaining positive founder mice via *Easi*-CRISPR can usually be achieved in fewer than three rounds of zygote injection (e.g., the positive mouse shown in Fig. 4D was generated upon the first injection round), and the lssDNA quantity procured via CRISPR-*CLIP* is sufficient for five or six rounds, demonstrating the practical usage of the method. In addition, the material cost incurred by lssDNA production appears to be comparable to that of the PCR-based method (∼$300 per *Easi*-CRISPR project, pricing based on Takara Bio kit). Better still, the CRISPR-*CLIP* skips the technical uncertainty associated with PCR.

**FIG. 6.**
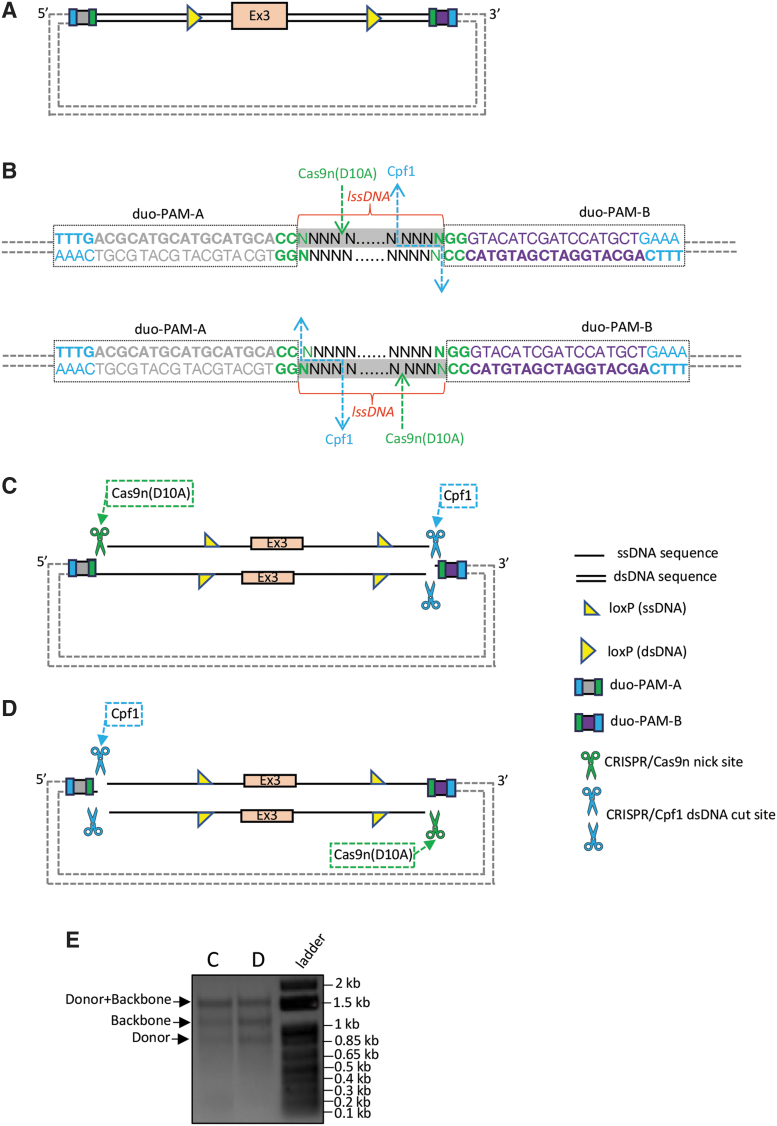
CRISPR-*CLIP* with add-on feature of universal DNA-tag sequences of Cpf1-Cas9 duo-PAM. **(A)** The dsDNA template carrying lssDNA^#^ cassette is flanked with duo-PAM-A (upstream end) and duo-PAM-B (downstream end), anchored in the default plasmid. **(B)** Each duo-PAM tag contains 23 bp sequence with PAMs for Cpf1 and Cas9 placed at respective 5′ ends of each DNA strand, plus a constant Cpf1 spacer sequence. Duo-PAM-A and -B are assigned with two distinct Cpf1 spacer sequences to enable suitable Cas types to make exclusive incisions on the plasmid at both ends of lssDNA junction sites. Of note, Cas9 spacer sequence is variable and subject to lssDNA donor; Cpf1 and Cas9 incisions on the duo-PAM tags will each remove 4 nt (end sequences of HA) from the lssDNA donor, which merely results in trivial variation in HA length. **(C)** To procure the sense ssDNA (top strand) as the lssDNA donor, choose Cas9n to cut at duo-PAM-A end while choosing Cpf1 to cut at duo-PAM-B end. **(D)** To procure the antisense ssDNA (bottom strand) as the lssDNA donor, choose Cpf1 to cut at the duo-PAM-A, while using Cas9n to cut at the duo-PAM-B. **(E)** Upon DGLB treatment, **(C)** and **(D)**, respectively, yielded three stand-alone ssDNA units of distinct sizes resolved on 0.9% agarose gel by electrophoresis. ^#^Belongs to a locus different from the case shown in Figures 4 and 5.

We have now routinely applied the CRISPR-*CLIP* method to generate donors that have been supplied in *Easi*-CRISPR for creating novel gene knock-in, humanization, CKO, and more recently for conditional knock-in models in mice (Supplementary Fig. S1A). The latter case carried a Lox66/71 floxing cassette, with Psen1 WT (exon 10) and the inversely positioned mutant (exon 10 with 15 bp AA replacement sequence) fused within, leading to a template 3.5 kbase long (Supplementary Fig. S1B and C and Supplementary Data Sequence 4). These CRISPR-*CLIP*-mediated donors collectively exemplify an effective approach to procure lssDNA templates encoding fairly large yet diverse genetic modifications for efficient generation of mouse models via *Easi*-CRISPR. That said, finding the prerequisite PAM for CRISPR-*CLIP* target sites for incisions on the dsDNA template plasmid may not always be possible, especially in Cpf1's case. To cope with such an issue, two add-on universal sequences of Cpf1-Cas9 can be placed on the plasmid at the exact junction sites flanking the lssDNA cassette (duo-PAM-A at upstream end; duo-PAM-B at downstream end; Fig. 6A), which also streamlines the donor design process. Specifically, duo-PAM-A carries both PAMs with a 19 nt Cpf1 spacer sequence,^23^ where Cpf1 and Cas9's PAMs are positioned at the 5′ end of the top and bottom strands, respectively, of the add-on DNA-tag, while duo-PAM-B carries both PAM sequences in inverse orientation of the duo-PAM-A (Fig. 6B and Supplementary Table S2). Moreover, both duo-PAM tags are assigned with distinct Cpf1 spacer sequences such that the DNA strand polarity of choice can be acquired by simply choosing suitable Cas type to make an exclusive type of incision on the plasmid at the particular end of the lssDNA cassette junction: use Cas9n to incise duo-PAM-A but Cpf1 to incise duo-PAM-B for acquiring the top strand (Fig. 6B and C) versus use Cpf1 to incise duo-PAM-A but Cas9n to incise duo-PAM-B for acquiring the bottom strand (Fig. 6B and D).

Further, the implementation of duo-PAM is reasoned to incorporate the provision for top versus bottom strand choice of the plasmid as a preferential lssDNA donor. A kinetic study reported long residency time of Cas9 on DNA double-stranded break target site with an asymmetric dissociation timeline from four broken strands, wherein the 3′ end of the cleaved DNA strand that is not complementary to the sgRNA (nontarget strand) is released first while the other three strands are still tethered to the Cas9–sgRNA complex.^24^ Such a scenario implicates accessibility lag among the cleaved DNA strands for initiating strand complementation in the DNA repair process. We hypothesize that supplying a lssDNA donor that carries a complementary sequence to the nontarget strand exploiting its PAM-distal (3′) end with immediate accessibility may potentially gain leverage in the recombination event, thereby enhancing the donor insertion rates. In this same context, extending the HA that is homologous to the PAM-proximal end longer than the PAM-distal end complementary HA aimed to offset possible ssDNA exonuclease degradation of the former HA. While this hypothesis warrants further investigation, the add-on duo-PAM feature provides feasibility for choosing the optimal strand of lssDNA donor when suitable, regardless of availability of prerequisite PAM(s) in the plasmid for CRISPR incision(s). Overall, the CRISPR-*CLIP* with universal duo-PAM tag further simplifies the lssDNA generation process and broadens its applicability to suit any genomic sequences of interest. Together with CRISPR-*CLONInG*, both methods illustrate strategies for efficient construction of highly customizable donor templates to facilitate genome editing.

## Supplementary Material

Supplemental data

Supplemental data

Supplemental data

## Supplementary Material

Supplementary Table S2

Supplementary Data Sequence 1

Supplementary Data Sequence 2

Supplementary Data Sequence 3

Supplementary Data Sequence 4

## Notes

^*^ All nucleic acid quantifications were performed by NanoDrop Spectophotometer.

^†^ To maintain confidentiality of the research, the name of the gene was altered.
