# Supplementary material for: New Additions to the CRISPR Toolbox: CRISPR-CLONInG and CRISPR-CLIP for Donor Construction in Genome Editing
Source: CRISPR J. 2020 Apr 21;3(2):109–22. doi: 10.1089/crispr.2019.0062 (PMC7194329; doi:10.1089/crispr.2019.0062)
Supplement: Supplemental data [file Supp_Table-S1-S2.pdf]

Supplementary Table S1. Primer Sequences for PCR Amplification of Vector Inserts and for Screening the Insertion of Psen1 AA Replacement Cassette in N2A Cell Line

Primer name	*Primer sequence*
**Neo-F**[a]	tgtntcncntcnnagnggantcntaATAAGCTTGATATCGAATTCCGAAGTTCCTA
**Neo-R**	gctgtacaagtagATATTATGTACCTGACTGATGAAGTTCCTATACTTTCTAG
**tdTom-F**	aggtacataatatCTACTTGTACAGCTCGTCCATGCCGTACAGGAACAGGTGG
**tdTom-R**	tttgaaaaacacgatgataatatggccacaACCATGGTGAGCAAGGGCGAGGAGGTCATCAAAGAGTT
**AAV-F**	GTACATCTACGTATTAGTCATCGCTATTAC
**AAV-R**	GGCCGCTCGGTCCGCACGT
**PSN1-F**	CTCTAGCTTGTGTCAGGTTGGTGTGGTA
**PSN1-R**	GGCAGCTCGGTGACACTTTGTC
**PSN1-seq**	ATAGCCAGACCGCATGC

[a]N indicates the nt that was changed to maintain research confidentiality.

Supplementary Table S2. CRISPR Guide RNAs and Binding Sites

gRNA name	*CRISPR target site (PAM in bold)*
**Luc-A**[a]	CNTCANAGTNGACTCNTACA**CGG**
**Luc-B**	TTTGGCATCTTCCATGGTTG**TGG**
**AAV-A**	CATCGCTATTACCATGGTCG**AGG**
**AAV-B**	CCCTGTCCTTCTGATTTTGT**AGG**
**CLIP-A (for Cas9)**[a]	TNCTNTTNGTNCTNGGNTAN**AGG**
**CLIP-B (for Cpf1)**[a]	**TTTN**CTANTTGNTCNAGGNAGNTTNAAN
**Duo-PAM-A**	**TTTG**ACGCATGCATGCATGCA**CC**
**Duo-PAM-B**	**TTTC**AGCATGGATCGATGTAC**CC**

[a]N indicates the nt that was changed to maintain research confidentiality.
