# Supplementary material for: New Additions to the CRISPR Toolbox: CRISPR-CLONInG and CRISPR-CLIP for Donor Construction in Genome Editing
Source: CRISPR J. 2020 Apr 21;3(2):109–22. doi: 10.1089/crispr.2019.0062 (PMC7194329; doi:10.1089/crispr.2019.0062)
Supplement: Supplemental data [file Supp_Data.pdf]

Sequence 1. CRISPR-*CLONInG*: Partial sequence of final FLEx vector with partial 3′UTR, FRT-PGK-Neo-FRT, tdTomato, and IRES sequences

3′UTR (gray highlight); FRT-PGK-Neo-FRT, inverted sequence (yellow highlight), tdTomato, inverted sequence (red highlight), IRES (blue highlight)

CNTCANAGTNGACTCNTAATAAGCTTGATATCGAATTCCGAAGTTCCTATTCTCTAGAAAGTATAGG
AACTTCAGGTCTGAAGAGGAGTTTACGTCCAGCCAAGCTAGCTTGGCTGCAGGTCGTCGAAATTCTAC
CGGGTAGGGGAGGCGCTTTTCCCAAGGCAGTCTGGAGCATGCGCTTTAGCAGCCCCGCTGGGCACTTG
GCGCTACACAAGTGGCCTCTGGCCTCGCACACATTCCACATCCACCGGTAGGCGCCAACCGGCTCCGT
TCTTTGGTGGCCCCTTCGCGCCACCTTCTACTCCTCCCCTAGTCAGGAAGTTCCCCCCCGCCCCGCAGCT
CGCGTCGTGCAGGACGTGACAAATGGAAGTAGCACGTCTCACTAGTCTCGTGCAGATGGACAGCACCG
CTGAGCAATGGAAGCGGGTAGGCCTTTGGGGCAGCGGCCAATAGCAGCTTTGCTCCTTCGCTTTCTGG
GCTCAGAGGCTGGGAAGGGGTGGGTCCGGGGGCGGGCTCAGGGGCGGGCTCAGGGGCGGGGCGGGCG
CCCGAAGGTCCTCCGGAGGCCCGGCATTCTGCACGCTTCAAAAGCGCACGTCTGCCGCGCTGTTCTCCT
CTTCCTCATCTCCGGGCCTTTCGACCTGCAGCCTGTTGACAATTAATCATCGGCATAGTATATCGGCAT
AGTATAATACGACAAGGTGAGGAACTAAACCATGGGATCGGCCATTGAACAAGATGGATTGCACGCA
GGTTCTCCGGCCGCTTGGGTGGAGAGGCTATTCGGCTATGACTGGGCACAACAGACAATCGGCTGCTC
TGATGCCGCCGTGTTCCGGCTGTCAGCGCAGGGGCGCCCGGTTCTTTTTGTCAAGACCGACCTGTCCGG
TGCCCTGAATGAACTGCAGGACGAGGCAGCGCGGCTATCGTGGCTGGCCACGACGGGCGTTCCTTGCG
CAGCTGTGCTCGACGTTGTCACTGAAGCGGGAAGGGACTGGCTGCTATTGGGCGAAGTGCCGGGGCAG
GATCTCCTGTCATCTCACCTTGCTCCTGCCGAGAAAGTATCCATCATGGCTGATGCAATGCGGCGGCTG
CATACGCTTGATCCGGCTACCTGCCCATTCGACCACCAAGCGAAACATCGCATCGAGCGAGCACGTAC
TCGGATGGAAGCCGGTCTTGTCGATCAGGATGATCTGGACGAAGAGCATCAGGGGCTCGCGCCAGCCG
AACTGTTCGCCAGGCTCAAGGCGCGCATGCCCGACGGCGATGATCTCGTCGTGACCCATGGCGATGCC
TGCTTGCCGAATATCATGGTGGAAAATGGCCGCTTTTCTGGATTCATCGACTGTGGCCGGCTGGGTGTG
GCGGACCGCTATCAGGACATAGCGTTGGCTACCCGTGATATTGCTGAAGAGCTTGGCGGCGAATGGGC
TGACCGCTTCCTCGTGCTTTACGGTATCGCCGCTCCCGATTCGCAGCGCATCGCCTTCTATCGCCTTCTT
GACGAGTTCTTCTGAGGGGATCAATTCTCTAGAGCTCGCTGATCAGCCTCGACTGTGCCTTCTAGTTGC
CAGCCATCTGTTGTTTGCCCCTCCCCCGTGCCTTCCTTGACCCTGGAAGGTGCCACTCCCACTGTCCTTT
CCTAATAAAATGAGGAAATTGCATCGCATTGTCTGAGTAGGTGTCATTCTATTCTGGGGGGTGGGGTG
GGGCAGGACAGCAAGGGGGAGGATTGGGAAGACAATAGCAGGCATGCTGGGGATGCGGTGGGCTCT
ATGGCTTCTGAGGCGGAAAGAACCAGCTGGGGCTCGACTAGAGCTTGCGGAACCCTTCGAAGTTCCTA
TTCTCTAGAAAGTATAGGAACTTCATCAGTCAGGTACATAATATCTACTTGTACAGCTCGTCCATGCCG
TACAGGAACAGGTGGTGGCGGCCCTCGGAGCGCTCGTACTGTTCCACGATGGTGTAGTCCTCGTTGTG
GGAGGTGATGTCCAGCTTGGTGTCCACGTAGTAGTAGCCGGGCAGTTGCACGGGCTTCTTGGCCATGT
AGATGGTCTTGAACTCCACCAGGTAGTGGCCGCCGTCCTTCAGCTTCAGGGCCTGGTGGATCTCGCCCT
TCAGCACGCCGTCGCGGGGGTACAGGCGCTCGGTGGAGGCCTCCCAGCCCATGGTCTTCTTCTGCATT
ACGGGGCCGTCGGGGGGGAAGTTGGTGCCGCGCATCTTCACCTTGTAGATCAGCGTGCCGTCCTGCAG
GGAGGAGTCCTGGGTCACGGTCACCAGACCGCCGTCCTCGAAGTTCATCACGCGCTCCCACTTGAAGC
CCTCGGGGAAGGACAGCTTCTTGTAATCGGGGATGTCGGCGGGGTGCTTCACGTACGCCTTGGAGCCG
TACATGAACTGGGGGGACAGGATGTCCCAGGCGAAGGGCAGGGGGCCGCCCTTGGTCACCTTCAGCTT
GGCGGTCTGGGTGCCCTCGTAGGGGCGGCCCTCGCCCTCGCCCTCGATCTCGAACTCGTGGCCGTTCAT
GGAGCCCTCCATGCGCACCTTGAAGCGCATGAACTCTTTGATGACGGCCATGTTGTTGTCCTCGGAGGA
GGCGGTGCCGGAGCTGCCGCTGCCGGTGCTGCCGGTGCCATGCCCCAGGAACAGGTGGTGGCGGCCCT
CGGAGCGCTCGTACTGTTCCACGATGGTGTAGTCCTCGTTGTGGGAGGTGATGTCCAGCTTGGTGTCCA
CGTAGTAGTAGCCGGGCAGTTGCACGGGCTTCTTGGCCATGTAGATGGTCTTGAACTCCACCAGGTAG
TGGCCGCCGTCCTTCAGCTTCAGGGCCTGGTGGATCTCGCCCTTCAGCACGCCGTCGCGGGGGTACAG
GCGCTCGGTGGAGGCCTCCCAGCCCATGGTCTTCTTCTGCATTACGGGGCCGTCGGGGGGGAAGTTGG
TGCCGCGCATCTTCACCTTGTAGATCAGCGTGCCGTCCTGCAGGGAGGAGTCCTGGGTCACGGTCACCA
GACCGCCGTCCTCGAAGTTCATCACGCGCTCCCACTTGAAGCCCTCGGGGAAGGACAGCTTCTTGTAAT
CGGGGATGTCGGCGGGGTGCTTCACGTACGCCTTGGAGCCGTACATGAACTGGGGGGACAGGATGTCC
CAGGCGAAGGGCAGGGGGCCGCCCTTGGTCACCTTCAGCTTGGCGGTCTGGGTGCCCTCGTAGGGGCG

GCCCTCGCCCTCGCCCTCGATCTCGAACTCGTGGCCGTTCATGGAGCCCTCCATGCGCACCTTGAAGCG
CATGAACTCTTTGATGACCTCCTCGCCCTTGCTCACCATGGTTGTGGCCATATTATCATCGTGTTTTTCA
AAGGAAAACCACGTCCCCGTGGTTCGGGGGGCCTAGACGTTTTTTAACCTCGACTAAACACATGTAA
AGCATGTGCACCGAGGCCCCAGATCAGATCCCATACAATGGGGTACCTTCTGGGCATCCTTCAGCCCC
TTGTTGAATACGCTTGAGGAGAGCCATTTGACTCTTTCCACAACTATCCAACTCACAACGTGGCACTGG
GGTTGTGCCGCCTTTGCAGGTGTATCTTATACACGTGGCTTTTGGCCGCAGAGGCACCTGTCGCCAGGT
GGGGGGTTCCGCTGCCTGCAAAGGGTCGCTACAGACGTTGTTTGTCTTCAAGAAGCTTCCAGAGGAAC
TGCTTCCTTCACGACATTCAACAGACCTTGCATTCCTTTGGCGAGAGGGGAAAGACCCCTAGGAATGC
TCGTCAAGAAGACAGGGCCAGGTTTCCGGGCCCTCACATTGCCAAAAGACGGCAATATGGTGGAAAAT
AACATATAGACAAACGCACACCGGCCTTATTCCAAGCGGCTTCGGCCAGTAACGTTAGGGGGGGGGGA
GGGAGAGGGG

Sequence 2. CRISPR-*CLONInG*: Partial FLEx vector sequence showing CRISPR-Cas9 allows precise excision of Luciferase gene from its adjacent 3′UTR and IRES sequences, with only 1 nt deviation

3′UTR (gray highlight), luciferase gene, inverted sequence (green highlight), IRES (blue highlight), PAM NGG (bold), CRISPR target site (underlined), slash (Cas9 cleavage site), gRNA Luc-A (red); partial sequence changed to maintain research confidentiality; gRNA Luc-B (violet)

CNTCANAGTNGACTCNT/ACA**CGG**CGATCTTGCCGCCCTTCTTGGCCTTAATGAGAATCTCGCGGATC
TTGCGGGCGTCCAACTTGCCGGTCAGTCCTTTAGGCACCTCGTCCACGAACACAACACCACCGCGCAG
CTTCTTGGCGGTTGTAACCTGGCTGGCCACATAGTCCACGATCTCCTTCTCGGTCATGGTTTTACCGTGT
TCCAGCACGACGACTGCGGCGGGCAGCTCGCCGGCATCGTCGTCGGGCAGGCCGGCGACCCCGGCGTC
GAAGATGTTGGGGTGTTGCAGCAGGATGCTCTCCAGTTCGGCTGGGGCTACCTGGTAGCCCTTGTATTT
GATCAGGCTCTTCAGCCGGTCCACGATGAAGAAGTGCTCGTCCTCGTCCCAGTAGGCGATGTCGCCGCT
GTGCAGCCAGCCGTCCTTGTCGATGAGAGCGTTTGTAGCCTCGGGGTTGTTAACGTAGCCGCTCATGAT
CATGGGGCCACGGACGCACAGCTCGCCGCGCTGGTTCACACCCAGTGTCTTACCGGTGTCCAAGTCCA
CCACCTTAGCCTCGAAGAAGGGCACCACCTTGCCTACTGCGCCAGGCTTGTCGTCCCCTTCGGGGGTGA
TCAGAATGGCGCTGGTTGTTTCTGTCAGGCCGTAGCCCTGGCGGATGCCTGGTAGGTGGAAGCGTTTGG
CCACGGCCTCACCTACCTCCTTGCTGAGCGGCGCCCCGCCGCTGGCGATCTCGTGCAAGTTGCTTAGGT
CGTACTTGTCGATGAGAGTGCTCTTAGCGAAGAAGCTAAATAGTGTGGGCACCAGCAGGGCAGATTGA
ATCTTATAGTCTTGCAAGCTGCGCAAGAATAGCTCCTCCTCGAAGCGGTACATGAGCACGACCCGAAA
GCCGCAGATCAAGTAGCCCAGCGTGGTGAACATGCCGAAGCCGTGGTGAAATGGCACCACGCTGAGG
ATAGCGGTGTCGGGGATGATCTGGTTGCCGAAGATGGGGTCGCGGGCATGACTGAATCGGACACAAGC
GGTGCGGTGCGGTAGGGCTACGCCCTTGGGCAATCCGGTACTGCCACTACTGTTCATGATCAGGGCGA
TGGTTTTGTCCCGGTCGAAGCTCTCGGGCACGAAGTCGTACTCGTTGAAGCCGGGTGGCAAATGGGAA
GTCACGAAGGTGTACATGCTTTGGAAGCCCTGGTAGTCGGTCTTGCTATCCATGATGATGATCTTTTGT
ATGATCGGTAGCTTCTTTTGCACGTTGAGGATCTTTTGCAGCCCTTTCTTGCTCACGAATACGACGGTG
GGCTGGCTGATGCCCATGCTGTTCAGCAGCTCGCGCTCGTTGTAGATGTCGTTAGCTGGGGCCACAGCC
ACACCGATGAACAGGGCACCCAACACGGGCATGAAGAACTGCAAGCTATTCTCGCTGCACACCACGAT
CCGATGGTTTGTATTCAGCCCATAGCGCTTCATAGCTTCTGCCAGCCGAACGCTCATCTCGAAGTACTC
GGCGTAGGTAATGTCCACCTCGATATGTGCGTCGGTAAAGGCGATGGTGCCGGGCACCAGGGCGTAGC
GCTTCATGGCTTTGTGCAGCTGCTCGCCGGCGGTCCCGTCTTCGAGTGGGTAGAATGGCGCTGGGCCCT
TCTTAATGTTTTTGGCATCTTCCATGG/TTG**TGG**CCATATTATCATCGTGTTTTTCAAAGGAAAACCACG
TCCCCGTGGTTCGGGGGGCCTAGACGTTTTTTAACCTCGACTAAACACATGTAAAGCATGTGCACCGA
GGCCCCAGATCAGATCCCATACAATGGGGTACCTTCTGGGCATCCTTCAGCCCCTTGTTGAATACGCTT
GAGGAGAGCCATTTGACTCTTTCCACAACTATCCAACTCACAACGTGGCACTGGGGTTGTGCCGCCTTT
GCAGGTGTATCTTATACACGTGGCTTTTGGCCGCAGAGGCACCTGTCGCCAGGTGGGGGGTTCCGCTGC
CTGCAAAGGGTCGCTACAGACGTTGTTTGTCTTCAAGAAGCTTCCAGAGGAACTGCTTCCTTCACGACA
TTCAACAGACCTTGCATTCCTTTGGCGAGAGGGGAAAGACCCCTAGGAATGCTCGTCAAGAAGACAGG
GCCAGGTTTCCGGGCCCTCACATTGCCAAAAGACGGCAATATGGTGGAAAATAACATATAGACAAACG
CACACCGGCCTTATTCCAAGCGGCTTCGGCCAGTAACGTTAGGGGGGGGGGAGGGAGAGGGG

Sequence 3. CRISPR-*CLONInG*: Sequence of assembled custom AAV-v2, comprising sgRNA-W, sgRNA-X, and mutant exon10 of Psen1.

ITR (gray highlight), U6 promoter (green highlight), spacer sequence for W and X (yellow highlight), Tracr RNA (underlined), HA (italics), three amino acid replaced (red, green, and blue)

CCTGCAGGCAGCTGCGCGCTCGCTCGCTCACTGAGGCCGCCCGGGCAAAGCCCGGGCGTCGGGCGA
CCTTTGGTCGCCCGGCCTCAGTGAGCGAGCGAGCGCGCAGAGAGGGAGTGGCCAACTCCATCACTAGG
GGTTCCTGCGGCCGCACGCGTGAGGGCCTATTTCCCATGATTCCTTCATATTTGCATATACGATACAAG
GCTGTTAGAGAGATAATTGGAATTAATTTGACTGTAAACACAAAGATATTAGTACAAAATACGTGACG
TAGAAAGTAATAATTTCTTGGGTAGTTTGCAGTTTTAAAATTATGTTTTAAAATGGACTATCATATGCT
TACCGTAACTTGAAAGTATTTCGATTTCTTGGCTTTATATATCTTGTGGAAAGGACGAAACACCGTCTA
ACGAGTGAAGACCCGGGTTTTAGAGCTAGAAATAGCAAGTTAAAATAAGGCTAGTCCGTTATCAACTT
GAAAAAGTGGCACCGAGTCGGTGCTTTTTTTCTAGAAGAGGGCCTATTTCCCATGATTCCTTCATATTT
GCATATACGATACAAGGCTGTTAGAGAGATAATTGGAATTAATTTGACTGTAAACACAAAGATATTAG
TACAAAATACGTGACGTAGAAAGTAATAATTTCTTGGGTAGTTTGCAGTTTTAAAATTATGTTTTAAAA
TGGACTATCATATGCTTACCGTAACTTGAAAGTATTTCGATTTCTTGGCTTTATATATCTTGTGGAAAG
GACGAAACACCGCATTCTAACGAGTGAAGACCGTTTTAGAGCTAGAAATAGCAAGTTAAAATAAGGC
TAGTCCGTTATCAACTTGAAAAAGTGGCACCGAGTCGGTGCTTTTTTGTTTTAGAGCTAGAAATAGCAA
GTTAAAATAAGGCTAGTCCGTAGCGCGTGCGCCAATTCTGCAGACAAATTCTAGAGGTACCCGTTACA
TAACTTACGGTAAATGGCCCGCCTGGCTGACCGCCCAACGACCCCCGCCCATTGACGTCAATAATGAC
GTATGTTCCCATAGTAACGCCAATAGGGACTTTCCATTGACGTCAATGGGTGGAGTATTTACGGTAAAC
TGCCCACTTGGCAGTACATCAAGTGTATCATATGCCAAGTACGCCCCCTATTGACGTCAATGACGGTAA
ATGGCCCGCCTGGCATTATGCCCAGTACATGACCTTATGGGACTTTCCTACTTGGCAGTACATCTACGT
ATTAGTCATCGCTATTACCATGG*GACAGTCACAGTGCTGGGCATAGCCAGACCGCATGCAGGGAACATGG*
AAGCCATTTCTTCCTTCCTTAAAAAGAGGAAATAAAGGAGAAAATGTCAGCCCTCCTAGACATTGCCACATCAT
AGTTCCTCCACTGGAGAACTTACATGGAGAAAAGGAATTCCTGTGCTTGCCTTAACTGATACTCCGCCCTCTG
GAACGTGGCGGCGATGGTCTAAGGATTAAGTGCTCTGATTAACCTTCTCTCTTAAAGGAGCGGAGAGAGAGA
CACAGGACAGTGGTTCTGGGAACGATGATGGTGGCTTCAGTGAGGAGTGGGAGGCCCAAAGAGACAGTCAC
*CTGGGGCCTCATCGCTCCACTCCCGAGTCAAGAGCTGCTGTCCAGGAACTTTCTGGG*GCTATTCTAGCAGGT
GAAGACCCGGAGGAAAGTACGTGCATCACCTCTGCCTGACATCACGGCGGTGACATCACGGCGGTCTGTCA
GTGGCAGGGTTATCTGTCTTTCAGAGGATGTTGAGTTTTTCTGGGGAAATAGCTTATTCAGGTGTTTGCCACAC
AAACATAAGAGGCTGAGTTTAATCTCCAGCACTGTGTGTGTGTGTGTGTGTGTGTGTGTGTGTGTGTGTGTGA
GTGTGTGTGTGTGTGTGTGTGTGTGTGTGTGAGTGCGTGCATGGCCTCAGACACCTGTGCTCCCAGCACTGG
GCAGGTATAGACAGGACGACCTCTGGGGCTTGCTGACCAGCCGCTCTAACTGAACCATTCCAGTTCCGTGTG
*CTATGCCAGCTCCAAAACTAAGCTAGAGAATGATTCAAGAAGACACCAGGC*TGTAGGTAACCACGTGCGGA
CCGAGCGGCCGCAGGAACCCCTAGTGATGGAGTTGGCCACTCCCTCTCTGCGCGCTCGCTCGCTCACT
GAGGCCGGGCGACCAAAGGTCGCCCGACGCCCGGGCTTTGCCCGGGCGGCCTCAGTGAGCGAGCGAG
CGCGCAGCTGCCTGCAGGGGCGCCTGATGCGGTATTTTCTCCTTACGCATCTGTGCGGTATTTCACACC
GCATACGTCAAAGCAACCATAGTACGCGCCCTGTAGCGGCGCATTAAGCGCGGCGGGTGTGGTGGTTA
CGCGCAGCGTGACCGCTACACTTGCCAGCGCCCTAGCGCCCGCTCCTTTCGCTTTCTTCCCTTCCTTTCT
CGCCACGTTCGCCGGCTTTCCCCGTCAAGCTCTAAATCGGGGGCTCCCTTTAGGGTTCCGATTTAGTGC
TTTACGGCACCTCGACCCCAAAAAACTTGATTTGGGTGATGGTTCACGTAGTGGGCCATCGCCCTGATA
GACGGTTTTTCGCCCTTTGACGTTGGAGTCCACGTTCTTTAATAGTGGACTCTTGTTCCAAACTGGAAC
AACACTCAACCCTATCTCGGGCTATTCTTTTGATTTATAAGGGATTTTGCCGATTTCGGCCTATTGGTTA
AAAAATGAGCTGATTTAACAAAAATTTAACGCGAATTTTAACAAAATATTAACGTTTACAATTTTATGG
TGCACTCTCAGTACAATCTGCTCTGATGCCGCATAGTTAAGCCAGCCCCGACACCCGCCAACACCCGCT
GACGCGCCCTGACGGGCTTGTCTGCTCCCGGCATCCGCTTACAGACAAGCTGTGACCGTCTCCGGGAG
CTGCATGTGTCAGAGGTTTTCACCGTCATCACCGAAACGCGCGAGACGAAAGGGCCTCGTGATACGCC
TATTTTTATAGGTTAATGTCATGATAATAATGGTTTCTTAGACGTCAGGTGGCACTTTTCGGGGAAATG
TGCGCGGAACCCCTATTTGTTTATTTTTCTAAATACATTCAAATATGTATCCGCTCATGAGACAATAAC
CCTGATAAATGCTTCAATAATATTGAAAAAGGAAGAGTATGAGTATTCAACATTTCCGTGTCGCCCTTA

TTCCCTTTTTTGCGGCATTTTGCCTTCCTGTTTTTGCTCACCCAGAAACGCTGGTGAAAGTAAAAGATGC
TGAAGATCAGTTGGGTGCACGAGTGGGTTACATCGAACTGGATCTCAACAGCGGTAAGATCCTTGAGA
GTTTTCGCCCCGAAGAACGTTTTCCAATGATGAGCACTTTTAAAGTTCTGCTATGTGGCGCGGTATTAT
CCCGTATTGACGCCGGGCAAGAGCAACTCGGTCGCCGCATACACTATTCTCAGAATGACTTGGTTGAG
TACTCACCAGTCACAGAAAAGCATCTTACGGATGGCATGACAGTAAGAGAATTATGCAGTGCTGCCAT
AACCATGAGTGATAACACTGCGGCCAACTTACTTCTGACAACGATCGGAGGACCGAAGGAGCTAACCG
CTTTTTTGCACAACATGGGGGATCATGTAACTCGCCTTGATCGTTGGGAACCGGAGCTGAATGAAGCC
ATACCAAACGACGAGCGTGACACCACGATGCCTGTAGCAATGGCAACAACGTTGCGCAAACTATTAAC
TGGCGAACTACTTACTCTAGCTTCCCGGCAACAATTAATAGACTGGATGGAGGCGGATAAAGTTGCAG
GACCACTTCTGCGCTCGGCCCTTCCGGCTGGCTGGTTTATTGCTGATAAATCTGGAGCCGGTGAGCGTG
GGTCTCGCGGTATCATTGCAGCACTGGGGCCAGATGGTAAGCCCTCCCGTATCGTAGTTATCTACACGA
CGGGGAGTCAGGCAACTATGGATGAACGAAATAGACAGATCGCTGAGATAGGTGCCTCACTGATTAAG
CATTGGTAACTGTCAGACCAAGTTTACTCATATATACTTTAGATTGATTTAAAACTTCATTTTTAATTTA
AAAGGATCTAGGTGAAGATCCTTTTTGATAATCTCATGACCAAAATCCCTTAACGTGAGTTTTCGTTCC
ACTGAGCGTCAGACCCCGTAGAAAAGATCAAAGGATCTTCTTGAGATCCTTTTTTTCTGCGCGTAATCT
GCTGCTTGCAAACAAAAAAACCACCGCTACCAGCGGTGGTTTGTTTGCCGGATCAAGAGCTACCAACT
CTTTTTCCGAAGGTAACTGGCTTCAGCAGAGCGCAGATACCAAATACTGTCCTTCTAGTGTAGCCGTAG
TTAGGCCACCACTTCAAGAACTCTGTAGCACCGCCTACATACCTCGCTCTGCTAATCCTGTTACCAGTG
GCTGCTGCCAGTGGCGATAAGTCGTGTCTTACCGGGTTGGACTCAAGACGATAGTTACCGGATAAGGC
GCAGCGGTCGGGCTGAACGGGGGGTTCGTGCACACAGCCCAGCTTGGAGCGAACGACCTACACCGAA
CTGAGATACCTACAGCGTGAGCTATGAGAAAGCGCCACGCTTCCCGAAGGGAGAAAGGCGGACAGGT
ATCCGGTAAGCGGCAGGGTCGGAACAGGAGAGCGCACGAGGGAGCTTCCAGGGGGAAACGCCTGGTA
TCTTTATAGTCCTGTCGGGTTTCGCCACCTCTGACTTGAGCGTCGATTTTTGTGATGCTCGTCAGGGGG
GCGGAGCCTATGGAAAAACGCCAGCAACGCGGCCTTTTTACGGTTCCTGGCCTTTTGCTGGCCTTTTGC
TCACATGT

Sequence 4. CRISPR-*CLIP*: Sequence of lssDNA donor source template (dsDNA) for Psen1-CKI allele.

EcoRV (gray highlight), Lox66 (green highlight), Exon 10 (yellow highlight), Exon 10 with three amino acid KI (blue highlight), Lox71 (pink highlight), three amino acid replaced—inverted orientation (red, green, and blue), Cas9n site (underlined; PAM bolded)

GATATCACGAGAGTCTCCTAGAGAAACAGGCTCTCCCTAGTAAGTACACGTAGGACTATTCACGGA
GCTGAAATCCCCCCCCCCCACACACACACACACACTGTGAGTTCAAGACTAACCCTGACTGTGTGGGGA
GATCTTGTCACAGAAAGGGGTGGGAGTGCAAGGGTCTCTTGTCCAGTATGTCCAGAATAACTGAAAA
CATTAGGTGGGAGTGCAGTCAAGGTTACACTAGGCGCGCCAGTAGTATTTCAGTGGCCTGGGTAATG
GTGACAACCAAGGACTGTGATACTGCAGAAGGCACTAAAGAAAACATCAGAAGCTCTGTCAGAAAG
AAGTGAAGAACAGGGTGGTGTAAAACTGAAGCAGAAAGGAGCTTTCTTAAAAAGACAAACCTTAGA
AAGTTGAAAAAGAAAAGTAAGATGTTAGGCCTAATTAAGGCTATAGGCCTAATTTAAAGTAAAATAA
ATTTTATCTTATATGCATACATATGTGTATTTATATTTATGTGTTTGAGTGTTTGGCCTACATGTATGT
AGTGCACATACATGGCTGGTACCTGTGGAGGTCAGCAGAGGACATCCGATGCCCTGGGCTTGGGTGC
TAGTGAGCCACCATGTGGACACTGGGAACAAACCCCAAGTCTGTTGGAAGAGCCCTCTCCAGCCCCA
CGTGTCATATTGAGCTAAAGAGAAAAGTCGTGACTGTCTGCCTTAATCAGGGTCACTATTGGGGTGA
AACCAAACCATCATGGCTAAAGAACCTTGGGAAGAAAGGGGTTTGTTCAGCTTACACTTCCACATCA
CTGTTGGTCAGCAAAGGAACTCAGGACAGGAACTCAAATAGGACAGGGACCTGGGGCAGCGGAGGG
CGCTGCTTACTGGCTTACTCCCCATGGCTGTCTCAGCCTGCTTTCTTTCTCTTGAACTTGTACCACCAG
CCCATGGATGTCCCACCCAGAATTGGCTGGGCTTTCCCCCATCGATTACTCTTTAAGAGTATGCCCTA
TAGGGCTGCCTTCAGCTCCAGCTCTATTGTGTGGAGGCATTTTCTCAATGGAGGGTCCCTCCTCCGTG
ATGACTCTAGCTTGTGTCAGGTTGGTGTGGTAGCCAGCACATTATTTCTTGTGTATTTTTGGTGAGTTA
ACAAATGCTGACTGATTCCCTAGGAGTGTGCTCAATGTTTTCCTAGGCTCTGGGGAGAGAGGACTGA
ACAAGATGCTTCCTGTTGGTGTTCACAAGGGTGCAGGTTCTTCACGATTCCTGCTCGCCACCTTTGAC
ATACTGGGTTGTCGGCTGCCCTCATTTCCCTGTGGTCCTGACTTAATTAAATAACTTCGTATAGCATAC

ATTATACGAACGGTAAGTCACAGTGCTGGGCATAGCCAGACCGCATGCAGGGAACATGGAAGCCATT
TCTTCCTTCCTTAAAAAGAGGAAATAAAGGAGAAAATGTCAGCCCTCCTAGACATTGCCACATCATA
GTTCCTCCACTGGAGAACTTACATGGAGAAAAGGAATTCCTGTGCTTGCCTTAACTGATACTCCGCCC
TCTGGAACGTGGCGGCGATGGTCTAAGGATTAAGTGCTCTGATTAACCTTCTCTCTTAAAGGAGCGGA
GAGAGAGACACAGGACAGTGGTTCTGGGAACGATGATGGTGGCTTCAGTGAGGAGTGGGAGGCCCA
AAGAGACAGTCACCTGGGGCCTCATCGCTCCACTCCCGAGTCAAGAGCTGCTGTCCAGGAACTTTCT
GGGAGCATTCTAACGAGTGAAGACCCGGAGGAAAGTACGTGCATCACCTCTGCCTGACATCACGGCG
GTGACATCACGGCGGTCTGTCAGTGGCAGGGTTATCTGTCTTTCAGAGGATGTTGAGTTTTTCTGGGG
AAATAGCTTATTCAGGTGTTTGCCACACAAACATAAGAGGCTGAGTTTAATCTCCAGCACTGTGTGTG
TGTGTGTGTGTGTGTGTGTGTGTGTGTGTGTGAGTGTGTGTGTGTGTGTGTGTGTGTGTGTGTGAGTGC
GTGCATGGCCTCAGACACCTGTGCTCCCAGCACTGGGCAGGTATAGACAGGACGACCTCTGGGGCTT
GCTGACCAGCCGCTCTAACTGAACCATTCCAGTTCCGTGTGCTATGCCAGCTCCAAAACTAAGCTAGA
GAATGATTCAAGAAGACACCAGGCATCGACGTCGTACGTCGTCGATGCCTGGTGTCTTCTTGAATCA
TTCTCTAGCTTAGTTTTGGAGCTGGCATAGCACACGGAACTGGAATGGTTCAGTTAGAGCGGCTGGTC
AGCAAGCCCCAGAGGTCGTCCTGTCTATACCTGCCCAGTGCTGGGAGCACAGGTGTCTGAGGCCATG
CACGCACTCACACACACACACACACACACACACACACACTCACACACACACACACACACACACACAC
ACACACACACACAGTGCTGGAGATTAAACTCAGCCTCTTATGTTTGTGTGGCAAACACCTGAATAAG
CTATTTCCCCAGAAAAACTCAACATCCTCTGAAAGACAGATAACCCTGCCACTGACAGACCGCCGTG
ATGTCACCGCCGTGATGTCAGGCAGAGGTGATGCACGTACTTTCCTCCGGGTCTTCACCTGCTAGAAT
AGCCCCAGAAAGTTCCTGGACAGCAGCTCTTGACTCGGGAGTGGAGCGATGAGGCCCCAGGTGACTG
TCTCTTTGGGCCTCCCACTCCTCACTGAAGCCACCATCATCGTTCCCAGAACCACTGTCCTGTGTCTCT
CTCTCCGCTCCTTTAAGAGAGAAGGTTAATCAGAGCACTTAATCCTTAGACCATCGCCGCCACGTTCC
AGAGGGCGGAGTATCAGTTAAGGCAAGCACAGGAATTCCTTTTCTCCATGTAAGTTCTCCAGTGGAG
GAACTATGATGTGGCAATGTCTAGGAGGGCTGACATTTTCTCCTTTATTTCCTCTTTTTAAGGAAGGA
AGAAATGGCTTCCATGTTCCCTGCATGCGGTCTGGCTATGCCCAGCACTGTGACTATAACTTCGTATA
ATGTATGCTATACGAACGGTACCGCGGAGGCATCGACGTTTGGCCTCTAGGCACCGTGGACACACAT
AGCACACCCCCTCGGTGCCCTTCTTTTAATACATGTACACAAAAGAGAGAGAGTGTGGTGAATTAAA
ATGATAAGCAGATAATCTGAACAGCGCCAAAGCAGGAAGGTGGGCATTGAGATTCTAGGGTCACAC
AGAATCTAAAGCAGAGAGAAGGTAAAGAAGGCAATCTTTCTGCGGCCTGAACTGTACACACCACTTA
CTGACTTCTCTTTCAGTGCGGGACAAAGTGTCACCGAGCTGCCCAGGCAGGCCTCACACTCCTGGTCT
TAAACTATCCTCCTCCTCAGACTTCTGAGTAGCTCAGAGTGCAGGCTCACACCACCCTGCCCAGCTTG
GGATCATAGCTTTGAATTATGAAATCACAGAGTACTT**GGG**
