# Supplementary material for: New Additions to the CRISPR Toolbox: CRISPR-CLONInG and CRISPR-CLIP for Donor Construction in Genome Editing
Source: CRISPR J. 2020 Apr 21;3(2):109–22. doi: 10.1089/crispr.2019.0062 (PMC7194329; doi:10.1089/crispr.2019.0062)
Supplement: Supplemental data [file Supp_Fig-S1.pdf]

Supplementary Data

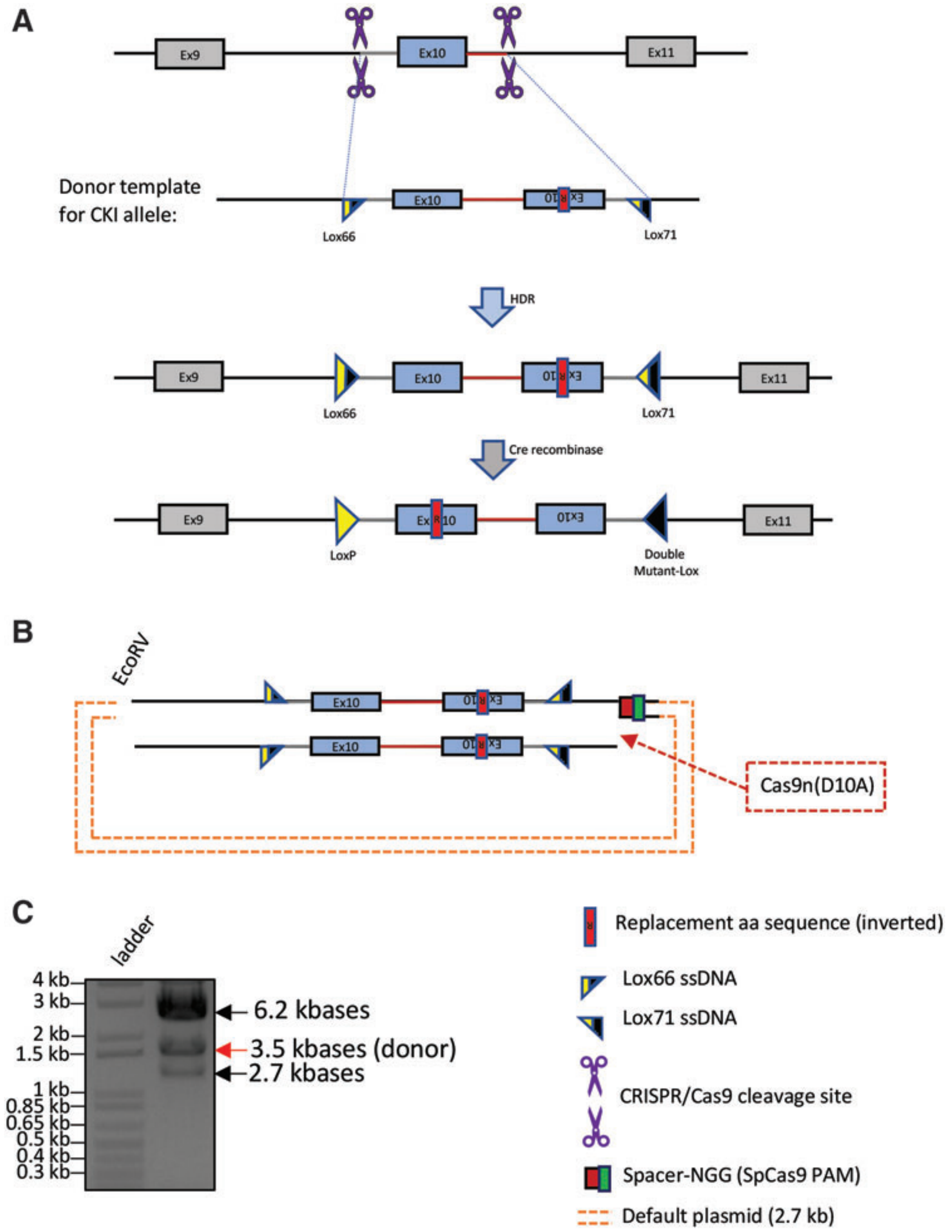

SUPPLEMENTARY FIG. S1. CRISPR-*CLIP*: Using Cas9n and RE (substitute for Cpf1) to generate 3.5 kbase lssDNA. **(A)** Schematic illustration of generating Psen1 conditional knock-in (CKI) allele. The donor template was designed to carry exon 10 and inverse mutant exon 10, flanked with Lox66 and Lox71 orientated in head-to-head position; once the donor is integrated into the target genome, the expression of Cre-recombinase catalyzes donor inversion to spatio-temporally express the mutant exon 10. **(B)** Plasmid containing assembled dsDNA template anchored in default backbone was incised by Cas9n and EcoRV with ssDNA and dsDNA cleavages, respectively, at respective ends of the dsDNA template. **(C)** The resulting three freestanding ssDNA units were treated with DGLB, followed by strand separation on a 0.9% agarose gel (arrows). The strand of interest (3.5 kbase lssDNA donor) was then clipped out via gel extraction and validated (data not shown).
